# Bionanocarbon Functional Material Characterisation and Enhancement Properties in Nonwoven Kenaf Fibre Nanocomposites

**DOI:** 10.3390/polym13142303

**Published:** 2021-07-14

**Authors:** Samsul Rizal, E. M. Mistar, A. A. Rahman, Abdul Khalil H.P.S., A. A. Oyekanmi, N. G. Olaiya, C. K. Abdullah, Tata Alfatah

**Affiliations:** 1 Department of Mechanical Engineering, Universitas Syiah Kuala, Banda Aceh 23111, Indonesia; 2 School of Industrial Technology, Universiti Sains Malaysia, Penang 11800, Malaysia; eka.marya.mistar@serambimekkah.ac.id (E.M.M.); abdulkan2000@yahoo.com (A.A.O.); ck_abdullah@usm.my (C.K.A.); tataalfatah83@gmail.com (T.A.); 3 School of Physics, Universiti Sains Malaysia, Penang 11800, Malaysia; 4 Department of Industrial and Production Engineering, Federal University of Technology, Akure PMB 704, Nigeria; ngolaiya@futa.edu.ng

**Keywords:** bionanocarbon, nanocomposite, X-ray density profile, activated carbon, characterisation, oil palm shell

## Abstract

Bionanocarbon as a properties enhancement material in fibre reinforced nanobiocomposite was investigated for sustainable material applications. Currently, an extensive study using the micro size of biocarbon as filler or reinforcement materials has been done. However, poor fibre-matrix interface results in poor mechanical, physical, and thermal properties of the composite. Hence in this study, the nanoparticle of biocarbon was synthesised and applied as a functional material and properties enhancement in composite material. The bionanocarbon was prepared from an oil palm shell, an agriculture waste precursor, via a single-step activation technique. The nanocarbon filler loading was varied from 0, 1, 3, and 5% as nanoparticle properties enhancement in nonwoven kenaf fibre reinforcement in vinyl ester composite using resin transfer moulding technique. The functional properties were evaluated using TEM, particle size, zeta potential, and energy dispersion X-ray (EDX) elemental analysis. While the composite properties enhancement was evaluated using physical, mechanical, morphological, thermal, and wettability properties. The result indicated excellent nanofiller enhancement of fibre-matrix bonding that significantly improved the physical, mechanical, and thermal properties of the bionanocomposite. The SEM morphology study confirmed the uniform dispersion of the nanoparticle enhanced the fibre-matrix interaction. In this present work, the functional properties of bionanocarbon from oil palm shells (oil palm industrial waste) was incorporated in nanaobiocomposite, which significantly enhance its properties. The optimum enhancement of the bionanocomposite functional properties was obtained at 3% bionanocarbon loading. The improvement can be attributed to homogeneity and improved interfacial interaction between nanoparticles, kenaf fibre, and matrix.

## 1. Introduction

Biocarbon has been proposed as a suitable material for mechanical strength and thermal enhancement for polymers due to its availability, thermal stability, and less energy requirement for its production. Biocarbon has been majorly produced from wood-based plants via thermal conversion, and this has a severe environmental impact on the forest reserves [1,2]. Recently, the isolation of biocarbon from biomass wastes has been extensively investigated by contemporary researchers [3,4,5]. This discovery has been proposed to reduce the overdependence on wood-based plants and petroleum products for its production [6]. Biomass such as palm kernel shell [7], coconut shell [8], sugarcane bagasse [9], and coffee straw [10] have been utilised for biocarbon production. Several techniques have been reported to produce biocarbon from biomass or biomass wastes [11]. The one-step activation method is energy-efficient and economically viable for the available techniques [12].

Natural fibre has a comparative advantage over synthetic fibre. It has been used as functional reinforcement material in composite because it is biodegradable, non-toxicity, and recyclable [13]. They are also low-density materials with relatively high strength and stiffness [14]. Due to their functional properties, a paradigm shift from conventional fibre reinforced composite to biofibre reinforced composite has been applied in studies. Natural fibre such as jute, sisal, hemp, wood, bamboo, and kenaf has been reported for reinforcements in polymer composite [15]. Applying kenaf fibre as a reinforcement agent has been widely reported in the literature due to its abundant nature, including its inherent properties such as high aspect ratio and superior toughness, non-abrasiveness, and biodegradability [16,17]. Due to its unique properties, Kenaf fibre has been utilised to reinforce composites applications [18,19,20]. Incorporating Kenaf fibre as filler in the matrix has been an efficient reinforcement agent for enhancing the interfacial interaction between fibre and matrix [19,21]. However, the challenge of low mechanical strength due to compatibility with hydrophobic polymer matrix and low thermal stability is yet to be addressed [18].

Vinyl ester has been extensively used as a matrix for liquid-moulding composite structures. The matrix offers attractive properties such as high toughness, low weight, low exothermal heat, low volume shrinkage, good chemical resistance, and relatively inexpensive [22]. Consequently, it has been used for various commercial applications such as surface vehicles, coatings, automobile parts, moulding compounds, adhesives, military/aerospace, and structural laminates [23]. Pure vinyl ester composite is brittle. A good approach to enhance the properties and reduce matrix cost is to strengthen with reinforcement and enhancement materials [24]. One approach for strengthening the vinyl ester is to mix it with bionanocarbon and kenaf fibre.

Mittal et al. [25] have reported that bio-based filler can enhance both thermal and mechanical properties of the synthesised composite. Recently, El Assami et al. [26] and Habti et al. [27] have reported the new concepts of the use of nanocarbon to improve the performances of structural composite materials. Remarkable attention has currently been devoted to developing nanoscale biomass material for many industrial applications [28]. No study has been done on the combined enhancement of nanocarbon and reinforcement of kenaf fibre in vinyl ester composites. The addition of bionanocarbon filler in nonwoven Kenaf fibre vinyl ester composites can give better mechanical and thermal stability to the fabricated composites [21].

Biocarbon nanoparticles were used to enhance the filler compatibility with hydrophobic polymer matrix and improve Kenaf fibre reinforced polymer composite mechanical and thermal stability. The nanosize of biocarbon was used due to its large surface area and the ability to improve the strength and compatibility of fibre-matrix interaction. The bionanocarbon was produced from oil palm shells (OPS) because it is one of the most generated solid wastes from the agricultural sector in the tropical region, producing a high percentage of biocarbon [29,30]. The present study aimed to develop and characterise the biocarbon nanoparticles used in combination with nonwoven kenaf fibre in vinyl ester composite. As functional nanomaterials were filled in Kenaf fibre, bionanocarbon was filled in reinforced vinyl ester composites via resin transfer moulding technique [31]. The activated bionanocarbon was incorporated in the composites at different filler loading. Characterisation studies on physical, mechanical, morphological, structural, and thermal properties of the activated bionanocarbon nonwoven kenaf fibre vinyl ester composites were investigated.

## 2. Materials and Methods

### 2.1. Materials

The (OPS) chips used in this study as the bionanocarbon precursor were collected from Ulu Keratong palm oil mill, Segamat, Johor, Malaysia. A nonwoven kenaf fibre mat with stitching and area density of 50 cm^−2^ and 1100 g/m^2^, respectively, was procured from Lembaga Kenaf dan Tembakau Negara, Kota Bharu, Kelantan, Malaysia. A needle-punched type of nonwoven kenaf fibres was used in this study. The fibre mat was cut according to the mould size (200 mm × 200 mm × 5.5 mm) and was used without further treatment. The microstructure, mechanical, chemical, thermal, and electrical properties of kenaf fibre are summarised in Table 1. Vinyl ester resin, methyl ethyl ketone peroxide (MEKP), and cobalt naphthenate were supplied by Zarm Scientific & Supplies Sdn. Bhd., Bukit Mertajam, Penang, Malaysia.

Other chemicals used in this study were of analytical grade, which includes potassium hydroxide (KOH), benzene (C_6_H_6_), toluene (C_7_H_8_), carbon tetrachloride (CCL_4_), hydrochloric acid (HCl), nitric acid (HNO_3_), acetic acid (CH_3_COOH), sodium hydroxide (NaOH), sodium carbonate (Na_2_CO_3_), and ammonium hydroxide (NH_4_OH). The practical grade of these chemicals was obtained from Sigma Aldrich, (St. Louis, MO, USA).

### 2.2. Preparation of Bionanocarbon from OPS

Chips of OPS were washed with deionised water and were then air-dried for 12 h to decrease the moisture to about 11%. The dried chips were crushed using a grinder equipped with a filter hole of 1.0 mm. The obtained particles were further ground using a Retsch mill with a filter of 0.25 mm. The grounded particles were sieved using 25 µm sieve size and were oven-dried at 110 °C for 24 h to eliminate inner moisture content before activation. The raw material was then soaked in (40% *w*/*v*) KOH solution with an impregnation ratio of 1:0.5 (mass of raw materials to KOH mass). The mixture was oven-dried at 120 °C for 4 h, followed by carbonisation at 700 °C for 30 min in a muffle furnace. The bio nanocarbon obtained was cooled to ambient temperature in a desiccator. It was later washed with 0.1M HCl solution at 85 °C, after which it was rewashed with hot distilled water until pH ~7 was achieved. Finally, the yielded bio nanocarbon was dried at 110 °C for 24 h. Nanostructured bionanocomposites were produced using high-energy ball milling (horizontal ball milling) at a rotation speed of 170 rpm for 24 h in an ambient environment. The stainless-steel chamber was loaded with a ratio of ball to carbon powder of 10:1 (*w*/*w*). The balls were made of stainless steel with 20 mm, 12 mm, and 10 mm diameter. After that, the bionanocarbon was oven-dried at 110 °C for 24 h and kept in a glass vial inside the desiccator.

### 2.3. Characterisation of Activated Bio Nanocarbon and Nonwoven Kenaf Fibre

Characterisation studies were carried out to investigate the properties of the prepared bio nanocarbon. The morphological properties of the synthesised materials were obtained using transmission electron microscopy (TEM), which was investigated with an energy-filtered EFTEM Libra 120—Carl Zeiss instrument (Oberkochen, Germany). Dynamic light scattering (DLS) was conducted on a Malvern Zetasizer Nano-ZS Ver. 7.11 (Malvern Instruments, Malvern, UK) for the evaluation of the particle sizes. Furthermore, the zeta potential measurement was conducted using a Malvern Zetasizer Nano Ver 7.11 (Malvern Instruments, Malvern, UK). The elemental composition of the activated bionanocarbon was analysed with Energy Dispersive X-ray (EDX) using field emission scanning electron microscope FESEM/EDX (FEI Quanta FEG 650, Thermo Fisher Scientific, Eindhoven, The Netherlands). The functional group analysis of bionanocarbon and nonwoven kenaf fibre was obtained using an FT-IR Prestige-21 spectrophotometer (Shimadzu, Chiyoda-ku, Tokyo, Japan). The morphologies of the nonwoven kenaf fibre were analysed using FESEM (EVO MA 10, Carl-ZEISS SMT, Oberkochen, Germany).

### 2.4. Preparation of Nanocomposites

The percentage ratio of vinyl ester resin to kenaf fibre was constant at 60:40 based on previous studies [38]. The activated bionanocarbon filler loading varies from 0, 1, 3, 5 wt% labelled as VE/K/NC0, VE/K/NC1, VE/K/NC3, and VE/K/NC5, respectively. The vinyl ester resin was mixed with 0.2 wt% of cobalt naphthenate and 1.5% wt% MEKP as an accelerator and a catalyst, respectively [39]. The vinyl ester resin-hardener mix was compounded with the bionanocarbon fillers and homogenised with a mechanical stirrer

**Figure 1:**
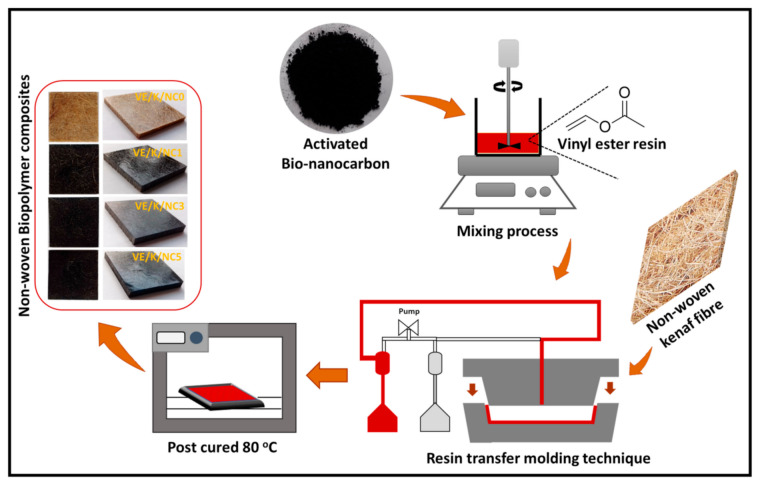
Schematic flow diagram of the nanocomposites’ preparation.

The samples were moulded with a Hypaject Mark II RTM injection system (Plastech, Thermoset Tectonics, Gunnislake, UK) connected to a 200 mm × 200 mm × 5.5 mm mould containing the kenaf fibre. The resin mix was injected into the mould at room temperature, a 200 kPa pressure and pressure gradient 1 kPa with vacuum assistance. The nanocomposites were cured at room temperature for 24 h and post-cured at 80 °C in an oven for 4 h. The obtained composites were cooled to ambient temperature in a desiccator containing granulated silica gel. Afterwards, they were cut to test samples and were placed in a zip lock bag and placed in a desiccator for analysis. The preparation of the flow chart of nanocomposites is illustrated in Figure 1.

### 2.5. Characterisation of Bionanocomposites

The physical properties of the fabricated bionanocomposites were obtained using density profile, wettability, and chemical resistance analysis. The density profile of the specimens with the thickness of bionanocarbon was investigated using X-ray Density Profiler Grecon model DA-X (Alfeld, Germany). The bionanocarbon composite samples with a 50 mm × 50 mm × 5.5 mm dimension were cut in a chamber at room temperature. The relative humidity of the sample before the investigation was 30 ± 2%. During scanning, the specimen was inserted into the cassette holder of each batch scan and analysed at 0.025 mm/s.

The test samples’ analysis of water absorption and swelling thickness measurements were investigated according to standard methods. The water absorption test aimed to study the water resistance capacity of the bio nanocomposite, while the thickness swelling test was conducted to evaluate the swelling of the samples. The tests were carried out according to ASTM D570 for all composite specimens [40]. Before the investigation, the weight and thickness of each specimen were recorded. Five test samples of bionanocomposite were immersed in distilled water at ambient atmosphere for 24 h. The samples were then taken out, and filter papers were used to remove the excess water on the surface before the weight and thickness were recorded. The rate of water absorption percentage was determined from Equation (1), and the percentage of thickness swelling was calculated according to Equation (2):

(1) $$\mathrm{Water}\;\mathrm{absorption}\;(\%)=\frac{W_{2}-W_{1}}{W_{1}}\;\times \;100$$

*W*_1_ is the weight of the samples before immersion, and *W*_2_ is the weight of samples after immersion:

(2) $$\mathrm{Thickness}\;\mathrm{swelling}\;(\%)=\frac{T_{2}-T_{1}}{T_{1}}\;\times \;100$$

where *T*_1_ and *T*_2_ are the thickness of the samples before and after soaking.

The morphological properties of the fractured surface after water absorption study were conducted to observe the effect of water absorption on the morphology of the bionanocomposite (WHY). The morphologies of the bionanocomposites were analysed using FESEM (EVO MA 10, Carl-ZEISS SMT, Oberkochen, Germany). A thin section of the sample was mounted on an aluminium (Al) stub holder with a double-sided copper (Cu) tape holder. The specimens were coated with a gold (Au) layer using sputter and coater Polaron SC515 to enhance their electrical conductivity. The FESEM micrographs were examined at an accelerating voltage of 10 kV under conventional secondary electron imaging stipulations.

The chemical resistance behaviour of the prepared bio nanocomposites was studied using the ASTM D543-87 method [41]. Studies were conducted to investigate the effect of solvents, acids, and alkalis on the bio nanocomposite. The investigated solvents, acid, and alkalis were (C_6_H_6_, C_7_H_8_, and CCL_4_), (HCl, HNO_3_, and CH_3_COOH), and (NaOH, Na_2_CO_3_, NH_4_OH), respectively. In each case, five pre-weighed samples were immersed in the respective chemical reagents for 24 h. The samples were removed and washed immediately with distilled water and dried by pressing the specimens on both sides with a filter paper at room temperature with precision (accuracy in ±0.01 mg). The percentage weight gain/loss was determined using the following Equation (3):

(3) $$\mathrm{Weight}\;\mathrm{gain}/\mathrm{loss}\;(\%)=\frac{\mathrm{Final}\;\mathrm{weight}\;-\;\mathrm{Original}\;\mathrm{weight}}{\mathrm{Original}\;\mathrm{weight}}\;\times \;100$$

The mechanical properties were analysed using tensile, flexural, and impact tests. The tensile strength, tensile modulus, and elongation at the breaking point of the prepared bio nanocomposites were measured using ASTM D638 specifications [42]. The flexural strength and modulus properties were investigated with the ASTM D790 standard [43]. The tensile and flexural tests were conducted using INSTRON 5582 universal testing (Norwood, MA, US). The impact strength was conducted using ASTM D256 for the Izod test [44]. The samples were notched at the centre before they were tested on a Gotech testing machine, Model GT-7045 MD (Taichung City, Taiwan). The five test specimens were evaluated, and the average values were recorded for each sample composition.

The functional group analysis of the bio nanocomposites was investigated using the Fourier transformation infrared (FT-IR). The analysis of test samples was obtained using an FT-IR Prestige-21 spectrophotometer (Shimadzu, Chiyoda-ku, Tokyo, Japan). The composite powder samples were prepared and oven-dried at 60 °C for 24 h before analysis was undertaken. The powder was mixed with KBR and pressed into the circular film before placing it in the FT-IR machine, and the results were obtained in transmittance. The spectra were obtained at the range of wavenumber from 4000–400 cm^−1^ at a resolution of 4 cm^−1^.

The thermal properties of the bio nanocomposites were examined using a Mettler–Toledo thermogravimetric analyser model TGA (Mettler Toledo, Schwarzenbach, Switzerland). About 10 mg of bionanocomposite was weighed in an alumina crucible and put in a thermogravimetric analyser with a pre-weighed empty alumina crucible as a reference. The thermal analysis was conducted within a temperature range of 30 °C to 800 °C, with a heating rate of 10 °C/min under nitrogen (N_2_) at a 50 mL/min flow rate. The results obtained were interpreted and derived through the STAR^e^ SW 10.00 software program for the evaluation of the onset temperature (T_on_) and maximum temperature (T_max_) of decomposition, including the mass loss (%).

The properties of the bio nanocomposites were determined through a comparative analysis using one-way ANOVA using DSAASTAT ver.1.101 by Andrea Onofri. The comparison between the mechanical and wettability properties of the bio nanocomposites was obtained at a probability coefficient of (*p* < 0.05) using Tukey’s HSD mean separation test.

## 3. Results and Discussion

### 3.1. Properties of Bio Nanocarbon and Kenaf Fibre

**Figure 2:**
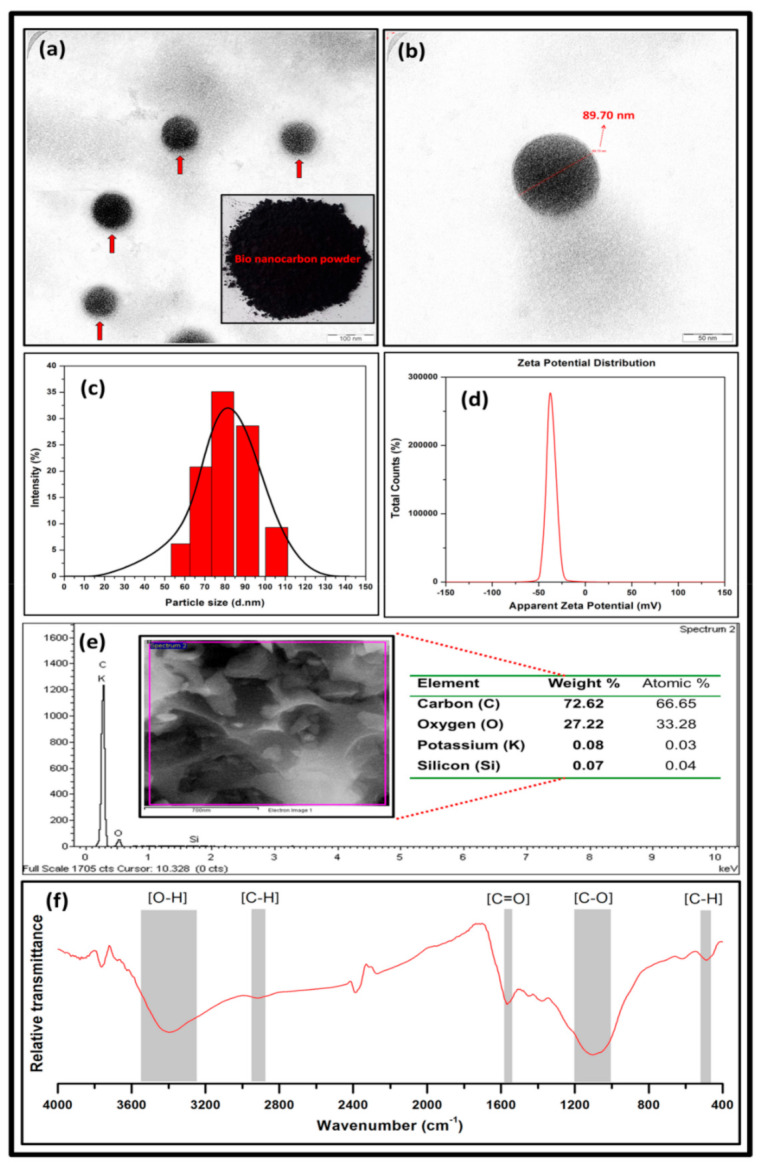
TEM micrograph (**a**) and nanosized powder (inset Figure) of bionanocarbon, (**b**) TEM micrograph of single nanoparticle of bionanocarbon, (**c**) particle size distribution of activated bionanocarbon, (**d**) potential zeta distribution of bionanocarbon, (**e**) elemental analysis of bionanocarbon, and (**f**) FT-IR spectra of bionanocarbon.

The morphological properties of the bionanocarbon were analysed using TEM images, which are presented in Figure 2a,b. The TEM morphology of the bio nanocarbon exhibited uniformly distributed particle size within 88 nm to 96 nm. As indicated in Figure 2a, the bionanocarbon exhibited black colouration without self-aggregation of powder. A noticeable particle size diameter of around 89.70 nm was observed in Figure 2b. The particle size distribution and zeta potential were illustrated in Figure 2c,d, respectively. It was denoted in Figure 2c that the particle diameter of the bionanocarbon was in the range of 59 nm to 106 nm with an average particle size of 81.4 nm. A trend of increase was indicated in particle diameter as the percentage intensity increases, although there was a very steep decrease in particle size diameter within 100 nm to 110 nm. As shown in Figure 2d, the zeta potential value of bionanocarbon was measured to be 35.6 ± 6.00 mV. The high estimated value could be attributed to the significance of the carboxyl functional group on the surface of the bionanocarbons [45]. The increase in surface densities of the functional groups such as hydroxyl and carboxyl is significant for the miscibility, compatibility, and dispersion stability of the filler in the matrix [46]. The zeta potential below −30 mV indicated the stability of the surface charge of the bio nanocarbon particles, which implies that sufficient mutual repulsion resulted in colloidal or emulsion stability [47].

The elemental analysis of the bionanocarbon was investigated using EDX. The elemental composition of the produced bionanocarbon is illustrated in Figure 2e. The area scan of the EDX spectrum indicated four noticeable peaks in the sample. The spectrum analysis results indicated that the bio nanocarbon was predominantly comprised of carbon (66.6%) and oxygen (33.3%) atoms; however, potassium, and silicon atoms were also present in small percentages. The significance of carbon and oxygen signifies that during KOH activation, the non-carbon component was decomposed, resulting in improved porosity and surface area of the bionanocarbon [48]. Similar findings from the previous study by Salehi and Hosseinifard [49] reported oxygen (40.2%) in nanocarbon to produce novel nanoporous carbon/polysaccharides nanocomposites. Moreover, the finding by Chen et al. [50] reported oxygen (38.5%) in nanocarbon to study oxygen content in the activated carbon. The value in this study was within this range in literature with a similar method. The FT-IR functional group analysis of the fabricated bionanocarbon is presented in Figure 2f. Five major adsorption peaks of bionanocarbon were noticed in the region 3500–3300 cm^−1^, 2950–2900 cm^−1^, 1600–1550 cm^−1^, 1200–1000 cm^−1^, and 500–450 cm^−1^, which indicated free –OH (hydroxyl), C–H (methyl), C=O (carboxyl), C–O (alkoxy) [51], and C–H (methyl) stretching bands, respectively [52]. Previous studies on FT-IR analysis of nanocarbon showed a similar functional group as reported by Lendzion-Bieluń et al. [51], Zhou et al. [53], and Kim et al. [52], respectively.

**Figure 3:**
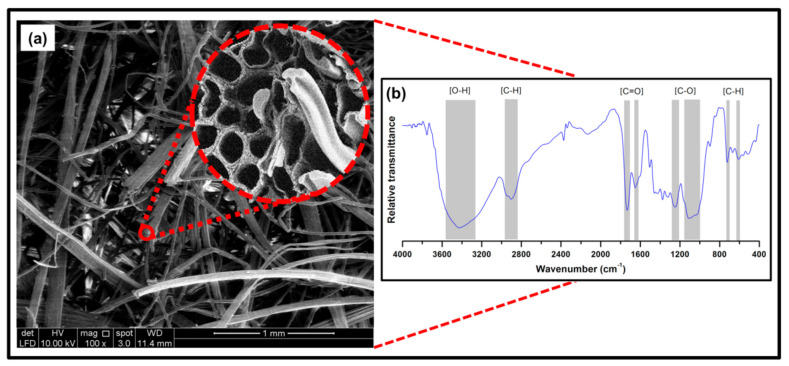
(**a**) FESEM micrograph and cross-section of nonwoven kenaf fibre, and (**b**) FT-IR spectra of nonwoven kenaf fibre.

The morphological and structural properties of the nonwoven kenaf fibre are illustrated in Figure 3. As shown in Figure 3a, the fibre mat structure possessed a linear and random structure of fibres as indicated in the cross-section, which signified the presence of porous interior structure Figure 3a, which could be illustrated in the corresponding fibres as exhibited by the grooved surface. FT-IR spectra of the nonwoven kenaf fibre are depicted in Figure 3b. The assigned broad region 3500–3250 cm^−1^ was associated with the O-H stretching vibration of hydroxyl groups. A band region about 2950–2850 cm^−1^ was ascribed to the asymmetric C–H representing stretching vibration in methyl groups. Region bands about 1775–1750 cm^−1^ and 1650–1625 cm^−1^ can be identified to the C=O carboxyl group. The peaks at 1250–1200 cm^−1^ and 1150–1000 cm^−1^ presence of C–O (alkoxy). The spectra around 725–600 cm^−1^ were attributed to the presence of C–H bend and cis.

### 3.2. Characterisation of Bionanocarbon/Kenaf Fibre/Vinyl Ester Composites

Characterisation studies were conducted to investigate the properties of the fabricated bionanocarbon/kenaf fibre/vinyl ester composites. The characterisation studies included investigating the physical, mechanical, fracture morphology, structural analysis, and the thermal properties of the synthesised bio nanocarbon/kenaf fibre/vinyl ester composites.

#### 3.2.1. Physical Properties

**Figure 4:**
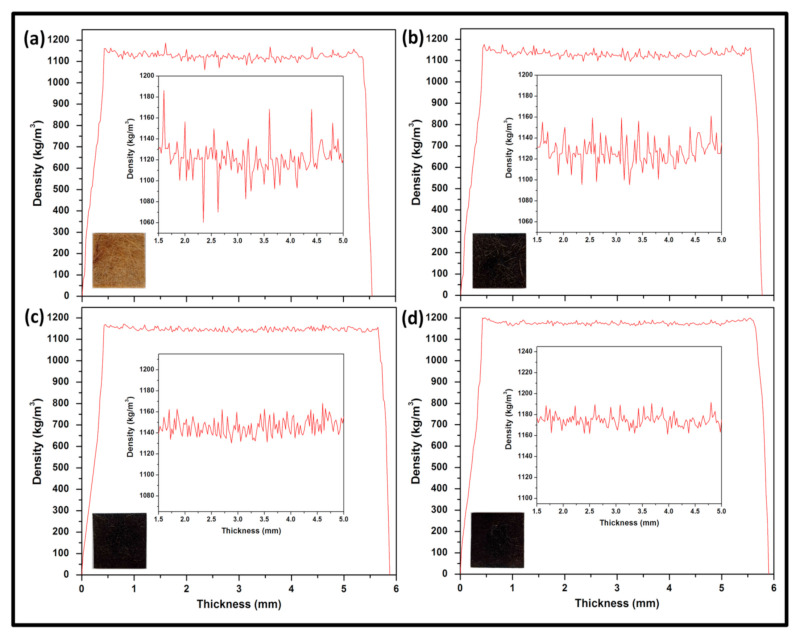
X-ray Density Profile of (**a**) VE/K/NC0 (**b**) VE/K/NC1(**c**) VE/K/NC3 and (**d**) VE/K/NC5.

The X-ray density profile of bio nanocomposites at different loading conditions is illustrated in Figure 4. Relative to the density of the control bionanocomposite (1072.19 kg/m^3^) in Figure 4a, it can be observed that the density of nanocomposite demonstrated a steady increase as filler loadings were increased probably because of the increase in compactness as a result of the large surface area of the nanoparticles. The highest density (1125.35 kg/m^3^) was achieved at VE/K/NC5 filler loading in Figure 4d. The stability of peak altitude and width influenced higher recorded density value, probably suggested that bionanofillers were distributed across the composite with no agglomeration or voids within the filler-matrix interaction. Therefore, the increase in the filler loading in composites causes a significant increase in polar groups at the surface; consequently, fibre-matrix bonding was improved [54]. Pati et al. [55] have investigated the filler effect in the matrix and concluded that the viscosity of the matrix was influenced by the filler loading, which increased as the filler increases. The results indicated that the viscosity stability within the fibre-matrix interface could be attributed to higher values of density of composite as loading was increased. It can be explained that the matrix permeated into voids and porous spaces within the filler to form interlocks within the composite materials.

The percentage water absorption and thickness swelling of the synthesised bio nanocomposites were achieved at *p* < 0.05. Table 2 summarises the water absorption, thickness swelling, and morphology of bio nanocomposite before and after immersion. The percentage of water absorption and thickness swelling decreased as filler loading increased relative to VE/K/NC0. As the filler loadings were increased, a more compact structure with closely aligned pores was observed in the composites’ surface. The filler loading at VE/K/NC3 indicated a more compacted structure of the composite. The moisture content and thickness swelling at this loading was 2.64 (±0.12) and 0.62 (±0.04), respectively. However, other loading resulted in noticeable voids that were predominant as filler loading was increased to VE/K/NC5. The filling of porous surfaces and voids with the nanofiller could probably improve the compatibility of fibre matrix interaction. The nanofiller in the interfacial region of the composite can reduce hydroxyl groups in the filler matrix interaction [56].

However, at VE/K/NC3 filler loading, the porosity of the composite porosity was significantly reduced due to a lack of voids for water absorption, resulting in a substantial decrease in the composite’s water absorption and thickness swelling. Beyond this filler loading, a slight increase in water absorption and thickness swelling was achieved. The moisture content and thickness swellings were 2.87 (±0.09) and 0.88 (±0.03) respectively at VE/K/NC5, indicating increased filler loading beyond VE/K/NC3 shows agglomeration within the fibre-matrix interface. The sample with 3 wt% nanocarbon gave very good miscibility. This means that below 3 wt% (1.5 wt%, 2.5 wt%) no agglomeration while above 3 wt% (3.5 wt% 4.5 wt% inclusive), there is the probability of agglomeration; thereby weak fibre-matrix interface [57,58]. The improvement of the fibre-matrix interaction compared to the control sample indicated that increased filler loading improved the compatibility.

A chemical resistance test was conducted to examine the suitability of the composite to chemical exposure. The test of these bionanocomposites was performed to determine whether they can be used to make them resistant to chemicals. The weight loss/gain for the bionanocomposites, including chemical resistance properties at different filler loading, is presented in Table 3. It is observed that the bionanocomposites did not lose weight, therefore erosion doesn’t seem to have occurred. It is indicated in comparative terms between the controlled nanocomposite (VE/K/NCO) and reinforced nanocomposite at different percentage filler loading. As filler loading increased and weight gain, a significant decrease in solvents was achieved optimally at VE/K/NC3 filler loading. A similar trend could be observed for the effect of acid and alkali resistance on nanocomposites which indicated that at VE/K/NC3, a firmly embedded structure impacted the filler-matrix interaction, consequently improved the surface properties of the nanocomposites in relation to chemical resistance. Moreover, the weight increase of the bionanocomposites was larger for aqueous solutions, which could be attributed to the hydrophilicity of the kenaf fibre. The incorporation of bionanocarbon probably could reduce the hydrophilicity of the system.

Statistical significance of the chemical resistance of nanocomposite was achieved at a confidence level of 95%, equivalent to *p* < 0.05, which indicated the aptness and suitability of the reinforced nanocomposite to chemical resistance.

#### 3.2.2. Mechanical Properties

**Figure 5:**
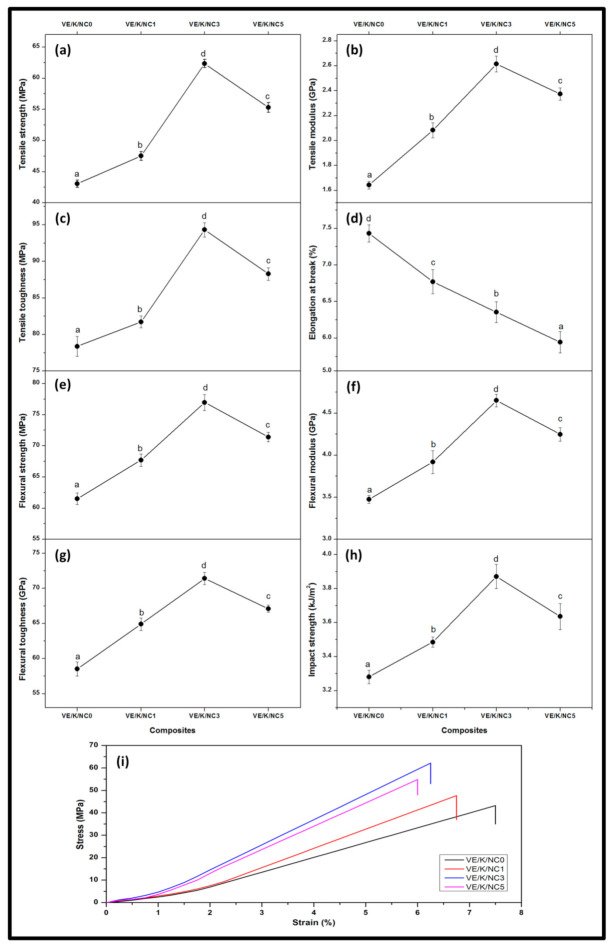
Mechanical properties of (**a**) tensile strength, (**b**) tensile modulus, (**c**) tensile toughness, (**d**) elongation at break, (**e**) flexural strength, (**f**) flexural modulus, (**g**) flexural toughness, (**h**) impact strength, and (**i**) stress-strain curves of nanocomposites. Values are presented as mean with one standard deviation error bar. The different superscript letters between data bars represent significant differences (*p* < 0.05).

The tensile properties of the bio nanocarbon/Kenaf fibre/vinyl ester composites are illustrated in Figure 5. The tensile strength and tensile modulus increased as the filler loadings were increased in the matrix. The highest tensile strength and tensile modulus were achieved at 3% filler loading at approximately 62.36 MPa and 2.61 GPa. A decrease in tensile strength and tensile modulus occurred due to increased filler loading [59]. This indicated the impact of activated biocarbon in the filler-matrix interaction, strongly related to the carboxyl and hydroxyl groups [52]. However, an inverse relationship was exhibited as tensile strength and tensile modulus were increased with filler loading, elongation at break decreased. Irrespective of the filler loading, the elongation at break decreased. The highest percentage decrease (5.94%) of elongation at break was achieved at VE/K/NC5 filler loading. The tensile toughness, flexural strength and flexural modulus are illustrated in Figure 5e–h. It is revealed that the incorporation of filler enhanced the mechanical properties of the bionanocomposites compared to controlled biocomposite without activated bionanocarbon. Percentage filler increase until VE/K/NC3 loading indicated the bionanocomposite exhibited an increase in the mechanical properties beyond 3% loading, an observable decrease in mechanical properties. At VE/K/NC3 filler loading, the highest tensile toughness (94.30 MPa), flexural strength (76.96 MPa), flexural modulus (4.65 GPa) were achieved.

A relative increment of the properties exhibited by the composites indicated that there was significant interfacial interaction between the filler within the matrix, especially the significance of bionanocarbon in the composite formulation. A progressive increase in tensile toughness, flexural strength, and flexural modulus as filler loading increased was probably due to the large surface area of the nanocarbon within the composite network between kenaf fibre and vinyl ester, resulting in a cohesive chain [60,61]. The effect of incorporating activated bionanocarbon as nanofiller on the impact strength of the bionanocomposite is illustrated in Figure 5h. The impact strength increased as filler loading increased. The highest impact was achieved at VE/K/NC3 loading, and the equivalent impact strength on composite was 3.87 kJ/m^2^. Incorporating optimum bionanocarbon as a functional nanofiller significantly enhanced the interfacial interaction between the reinforcing agent and the matrix, resulting in improved mechanical properties of bionanocomposites. Nanofiller improved the interfacial behaviour as it enhanced interlocking due to high surface area [62]. The influence of the nanofiller surface improved strong adhesion between the matrix and the reinforcement agent [63]. However, beyond VE/K/NC3 loading, a reduction of the impact strength of the composite was achieved, as shown in Figure 5i.

The tensile properties were evaluated using one-way ANOVA analysis to determine the statistical significance between the average tensile strength, tensile modulus, elongation at break, flexural strength, flexural modulus, flexural toughness, and impact strength of the bio nanocomposites at different loading conditions. A *p*-value less than 0.05 was achieved in all cases, suggesting that at a 95% confidence level, the synthesised bio nanocomposites exhibited good mechanical properties.

**Figure 6:**
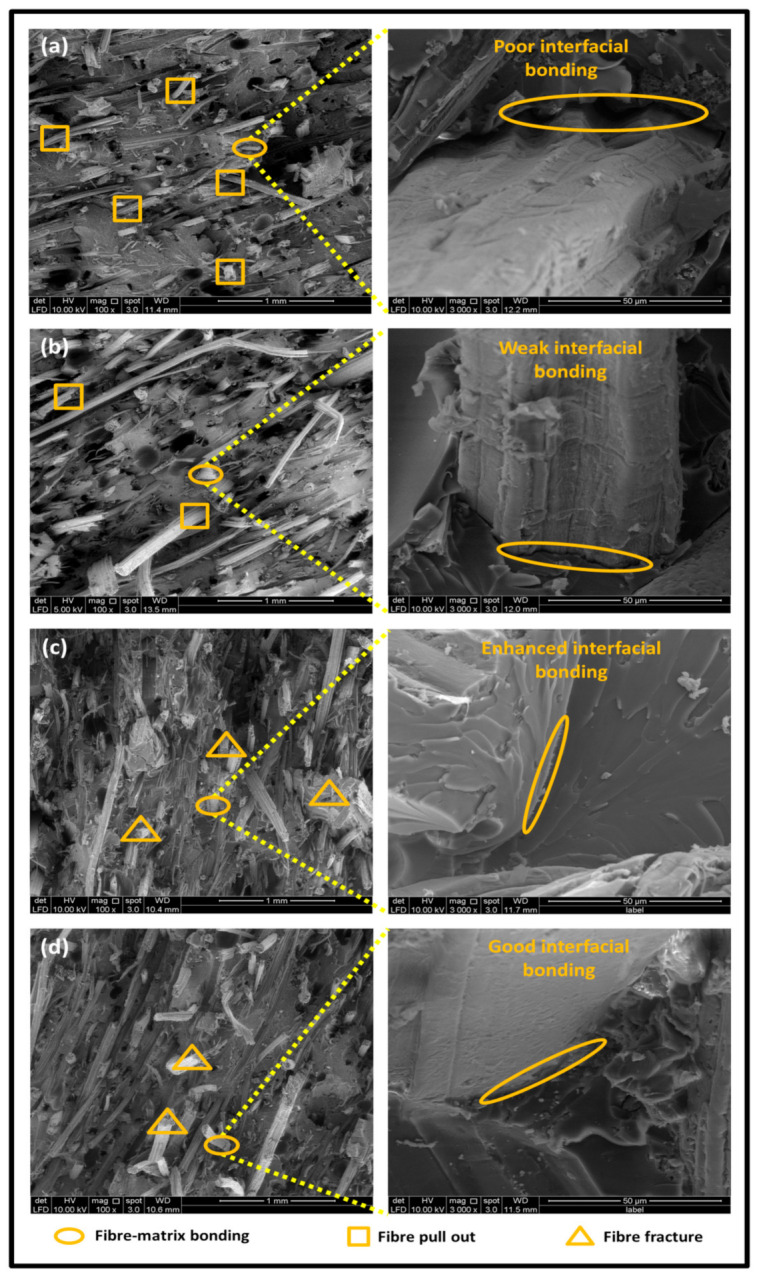
FESEM micrograph of the tensile fracture surface of (**a**) VE/K/NC0, (**b**) VE/K/NC1, (**c**) VE/K/NC3, and (**d**) VE/K/NC5.

The tensile fracture morphology of bionanocomposites at different bionanocarbon loading is presented in Figure 6. The compactness and miscibility of the biocomposite were observed to increase with nanocarbon loading. This is observed in the disappearance of void in the micrograph, as shown in Figure 6a–d. As observed in Figure 6a, holes and interfacial voids were seen in the SEM images. However, the addition of 1% loading of bionanocarbon lesser space between the material blends. The enhanced compactness is probably responsible for the increase in the tensile strength of the bionanocarbon. Previous studies show that interfacial defects could result from the immiscibility of the filler and tend to affect the interfacial bonding in the composite [20]. Therefore, incorporating bionanocarbon as filler in kenaf fibre reinforced vinyl ester composite results in a uniformly filled composite, as shown in Figure 6b–d.

In general, SEM morphology has shown improved fibre-matrix bonding as bionanocarbon loading increased compared with the control sample. At 1% filler loading, the fibre matrix bonding improved as compared to without bionanocarbon loading (Figure 6b). An increased fibre-matrix interaction was observed at 3% bionanocarbon loading (Figure 6c) due to enhanced interfacial bonding between bionanocarbon-kenaf fibre-matrix. From the SEM morphology, tensile fracture showed enhanced interfacial bonding due to the possibility of uniform dispersibility and highly homogenous nanofiller as enhancement in kenaf fibre-matrix bionanocomposites, indicating a cohesive chain in the interaction of fibre-matrix. The reduction in the mechanical properties of 5% loading may probably be due to the increased bionanocarbon percentage resulting in agglomeration, hence a decrease in the fibre-matrix bonding.

**Figure 7:**
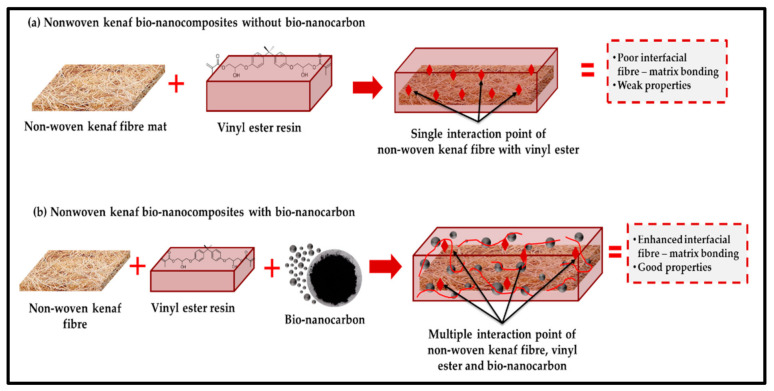
Possible interaction mechanism of nonwoven kenaf fibre nanocomposites with and without bionanocarbon.

The possible mechanism of the interaction between bionanocarbon and kenaf reinforcing fibre in vinyl ester bionanocomposite resulting in enhanced interfacial bonding is illustrated in Figure 7. It was indicated that the composite without bionanocarbon had poor interfacial bonding. On the other hand, the composite having low bionanocarbon loading (1%) exhibited an increase in the interfacial bonding despite weak bonding observed. Incorporating bionanocarbon with optimum percentage loading (3%) in kenaf fibre-vinyl ester promoted enhanced interfacial bonding of the composites. It provided uniform dispersibility and was highly homogenous across the available voids interface, which increased compatibility. However, the incorporation of bionanocarbon at high percentage loading decreased the interfacial bonding due to the agglomeration as explained in the previous section. The mechanism illustrated the incorporation of bionanocarbon in the kenaf fibre-vinyl ester bionanocomposites.

#### 3.2.3. Structural and Thermal Analysis

**Figure 8:**
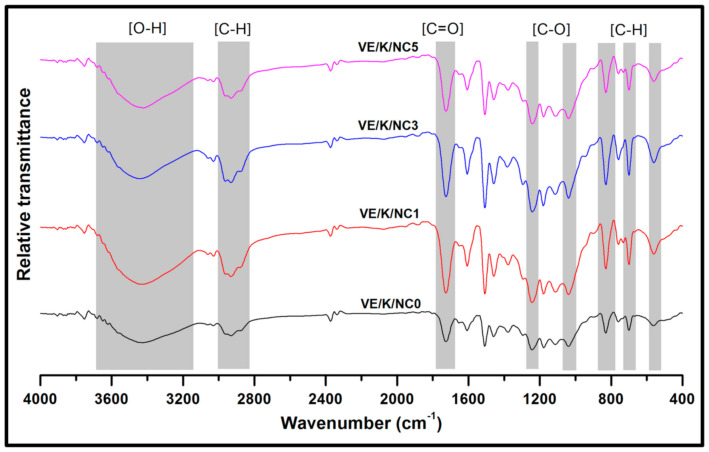
FT-IR spectra of nanocomposites with increasing activated bionanocarbon content.

The FT-IR spectra are presented in Figure 8. The predominance of functional groups was located in the region 3600–3200 cm^−1^, 3000–2800 cm^−1^, 1700–1800 cm^−1^, 1250 cm^−1^ and 850 cm^−1^ representing the free –OH, C–H, C=O, C–O, and C–H stretching bands, respectively. A slight increase in the –OH spectra as the filler loading increased indicated the hydroxyl group present in the bionanocarbon, as shown in its FT-IR analysis. [64]. The FT-IR analysis for the bionanocarbon, Kenaf fibre, and the bionanocomposite indicated that the active functional groups influenced the compatibility of the fibre-matrix interaction. However, no new functional group was formed other than those present in the constituent material, indicating that the significant increase in the properties is due to interfacial adhesion caused by the large surface area of the bionanocarbon and the binding properties of the properties matrix. This confirms the interfacial miscibility between the three components.

**Figure 9:**
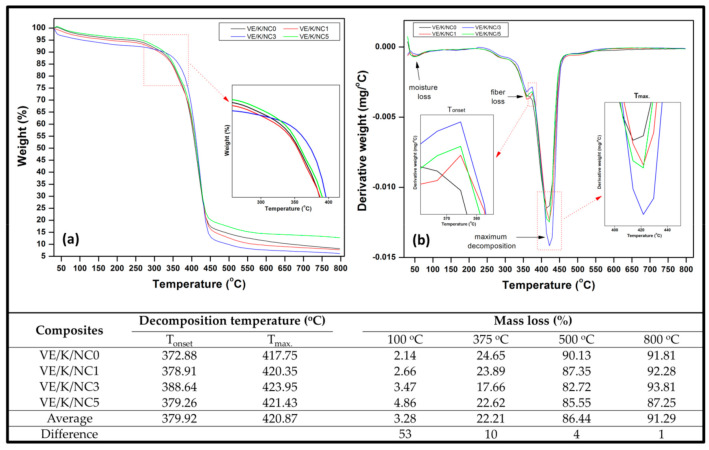
Thermogravimetric analysis of nanocomposites (**a**) TGA, (**b**) DTG, decomposition temperature and mass loss data of nanocomposites at different loading.

The thermal degradation properties of bio nanocomposites were analysed using thermogravimetric analysis (TGA) and derivative thermogravimetric (DTG) analysis presented in Figure 9. The TG curve indicated that initial weight loss occurred due to evaporation of absorbed water as this occurs below 100 °C. The first, second, and third degradation occurred between 290–310 °C, 310–360 °C, and 370–430 °C, respectively [62]. The first degradation was observed to be insignificant, which can probably be attributed to the degradation of other volatile substances present in the bionanocomposite based on literature, kenaf fibre, vinyl ester, and nanocarbon are relatively not decomposed at this temperature [39,52,65]. It could also be attributed to initial fibre loss of the nonwoven kenaf [20,65]. The third degradation is the bionanocarbon filled kenaf fibre vinyl ester bionanocomposite. The bionanocarbon and vinyl ester resin can withstand high temperatures. At this stage, maximum weight loss was achieved due to the degradation, and this is shown in the percentage weight loss tabulated in Figure 9. As tabulated in Figure 9, the onset temperature shows that its value increases with bionanocarbon loading to 3% and decreased by 5%. However, the 3% loading have the highest weight lost at 800 °C. Based on the DTG result; the peak temperature increases with bionanocarbon filler loading with 3% filler loading as the highest at 423.95 °C. Previous studies by Kim et al. [52] indicated that the addition of bionanocarbon provided thermal stability to the composite.

A summary of the numerical data obtained from the thermogravimetric temperature profile is illustrated in Figure 9. It is indicated that the temperature of decomposition (T_onset_) and maximum temperature of decomposition (T_max_) exhibited a noticeable shift to a higher temperature as the filler loading increased. There was a slight increase as indicated in the percent mass loss from 800 °C as the filler loading increased, compared to the control. There was a decrease in mass load beyond 3% filler loading. The T_onset_ shifted from 372.88 to 378.91 °C after the incorporation of 1% filler loading. Similarly, the temperature at which maximum degradation (T_max_) occurred shifted from 417.75 to 420.35 °C after 1% filler loading. This indicated that the incorporated nanofiller enhanced the thermal stability of biocomposite since high thermal stability is achieved at higher thermal decomposition temperature [65]. The T_onset_ and T_max_ proportionately increased as the filler loading increased from VE/K/NC1 to VE/K/NC1 with an average decomposition temperature of 379.92 and 420.87 °C, respectively.

Furthermore, optimum T_onset_ and T_max_ were achieved at VE/K/NC3 filler loading, indicating that optimum thermal stability of the composite occurred at 388.64 and 423.95 °C, which represented T_onset_ and T_max_, respectively. Incorporating the nanofiller further probably improved the compatibility of the composite structure by occupying the empty voids in the filler-matrix interface. Beyond VE/K/NC3 filler loading, a reduction in the thermal stability of the composite was observed. However, irrespective of the filler loading, mass loss increased at every stage of degradation temperature. The mass loss at 100 °C indicated that the effect of surface evaporation was about 0.52% less than the control at VE/K/NC1 loading. At this degradation temperature relative to the controlled composite, mass loss increased as filler loading increased. At elevated decomposition temperature, average mass loss decreased as compared to the control. This indicated that dehydroxylation due to the filled voids improved the inherent hydrophilic property of kenaf fibre in the matrix due to activated bio nanocarbon in the composite interface.

## 4. Conclusions

Bionanocomposites were synthesised via a resin transfer moulding technique. Activated bionanocarbon from oil palm shell precursor was produced using a single-step activation technique, and was applied to enhance vinyl nonwoven kenaf fibre composite. The nanocomposite’s physical, mechanical, morphological, and thermal properties were investigated using the TEM, FESEM, FT-IR, and TGA. From the physical property test, it can be concluded that the optimum density was achieved at VE/K/NC5 filler loading at a 95% confidence level. Furthermore, the nanocomposites’ water absorption and thickness swelling decreased as the filler loading increased with an optimal decrease in water absorption and thickness swelling achieved at VE/K/NC3 filler loading. It can be concluded from the result of the chemical resistance test revealed that the reinforced nanocomposites exhibited resistance to all chemicals used with less weight gain at a statistical significance of *p* < 0.05. The effect of filler loading reinforcement and enhancement indicated that at 3% filler loading, improved mechanical and thermal stability of nanocomposite was achieved, indicating the suitability of the reinforcement material and the nanofiller for improved composites applications. The incorporation of bionanocarbon generated from agriculture waste significantly improves the nanobiocomposite properties. The bionanocarbon also as an alternative to the man-made raw material in composite manufacturing will reduce composite production costs. Hence, the composite material produced in this study has potential for use in industrial applications such as automotive interior, furniture, and packaging.

## Figures and Tables

**Table 1:** The microstructure, mechanical, chemical, thermal, and electrical properties of kenaf fibre.

Property	Unit	Value
***Kenaf fibre***		
*Microstructure* [32]		
Number of the cells	-	21
Area	µm^2^	3678
Perimeter	µm	228.5
Major diameter	µm	89.03
Minor diameter	µm	56.7
*Mechanical* [33]		
Density	g/cm^3^	1.50
Tensile strength	MPa	930
Tensile modulus	GPa	53
Elongation	%	1.6
*Chemical* [34]		
Cellulose	%	56.45 ± 1.36
Hemicellulose	%	25.26 ± 0.65
Lignin	%	16.76 ± 1.56
*Thermal* [35]		
Initial degradation temperature	°C	338
Maximum degradation temperature	°C	346
*Electrical* [36,37]		
Surface resistivity	Ω/sq	1.2 × 10^10^
Conductivity	S/cm	7.51 × 10^−5^
***Vinyl ester***		
Appearance	-	Yellowish liquid
Viscosity [Brookfield @ 25 °C; Spindle #3 at 60 rpm]	cps	400–600
Acid value	-	<10
Styrene monomer content	%	42–45
Gelation time [Co 6%, 0.3%, MEKPO 55%, 1% at 30 °C]	minute	30–35

**Table 2:** The water absorption, thickness swelling, and FESEM images before and after immersion of nanocomposites (scale bar: 20 µm).

Composites	Water Absorption (%)	Thickness Swelling (%)	Before Immersion	After Immersion
VE/K/NC0	3.82 (±0.10) ^a^	1.39 (±0.07) ^a^	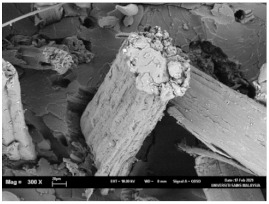	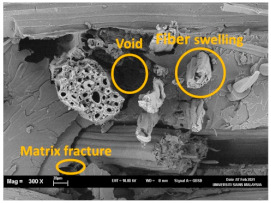
VE/K/NC1	3.23 (±0.11) ^b^	1.16 (±0.05) ^b^	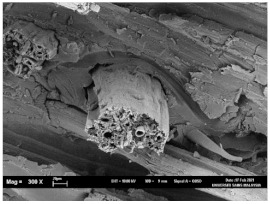	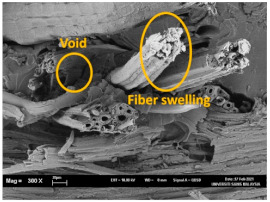
VE/K/NC3	2.64 (±0.12) ^d^	0.62 (±0.04) ^d^	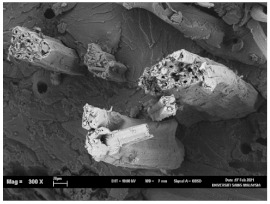	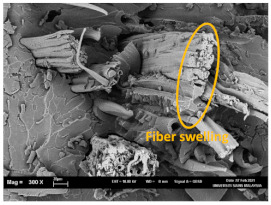
VE/K/NC5	2.87 (±0.09) ^c^	0.88 (±0.03) ^c^	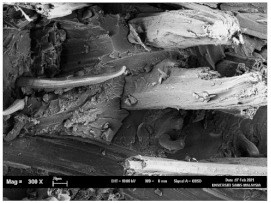	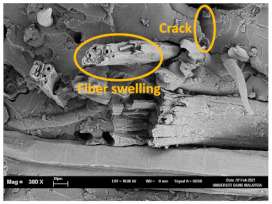

Values are presented as the mean with one standard deviation error bar. The same column followed by different superscript a, b, c, d letters indicates significant differences (*p* < 0.05).

**Table 3:** Chemical resistance properties of different loading of activated bionanocarbon reinforced nanocomposites.

Weight Gain (+)/Loss (−) (%)				
Chemicals	VE/K/NC0	VE/K/NC1	VE/K/NC3	VE/K/NC5
C_6_H_6_	4.06 ± 0.17 ^a^	2.75 ± 0.08 ^b^	0.38 ± 0.02 ^d^	1.43 ± 0.06 ^c^
C_7_H_8_	3.81 ± 0.12 ^a^	2.79 ± 0.06 ^b^	0.32 ± 0.01 ^d^	1.70 ± 0.08 ^c^
CCL_4_	3.97 ± 0.14 ^a^	2.48 ± 0.09 ^b^	0.29 ± 0.01 ^d^	1.61 ± 0.07 ^c^
HCl	9.64 ± 0.39 ^a^	7.03 ± 0.27 ^b^	1.47 ± 0.09 ^d^	3.85 ± 0.18 ^c^
HNO_3_ (40%)	8.30 ± 0.33 ^a^	6.57 ± 0.21 ^b^	1.28 ± 0.07 ^d^	3.48 ± 0.11 ^c^
CH3COOH (5%)	14.17 ± 0.58 ^a^	11.64 ± 0.35 ^b^	2.53 ± 0.08 ^d^	5.73 ± 0.25 ^c^
NaOH (10%)	11.85 ± 0.46 ^a^	7.97 ± 0.24 ^b^	3.46 ± 0.14 ^d^	6.14 ± 0.30 ^c^
Na_2_CO_3_ (20%)	8.92 ± 0.22 ^a^	6.10 ± 0.16 ^b^	0.71 ± 0.04 ^d^	1.82 ± 0.13 ^c^
NH_4_OH (10%)	16.43 ± 0.63 ^a^	10.85 ± 0.38 ^b^	3.19 ± 0.12 ^d^	5.67 ± 0.24 ^c^

Values are plotted as the mean with one standard deviation error bar. Mean in the same rows followed by different superscript a, b, c, d letters indicate significant differences (*p* < 0.05).

## Data Availability

The data presented in this study are available on request from the corresponding author.
